# The Unique Microbiome and Innate Immunity During Pregnancy

**DOI:** 10.3389/fimmu.2019.02886

**Published:** 2019-12-17

**Authors:** Chunlei Mei, Weina Yang, Xin Wei, Kejia Wu, Donghui Huang

**Affiliations:** ^1^Institute of Reproductive Health, Tongji Medical College, Huazhong University of Science and Technology, Wuhan, China; ^2^Second Affiliated Hospital of Jinlin University, Changchun, China; ^3^Zhongnan Hospital, Wuhan University, Wuhan, China

**Keywords:** pregnancy, microorganisms, innate immunity, maternal-fetus interface, NK cells, placenta, defense

## Abstract

A successful pregnancy depends on not only the tolerance of the fetal immune system by the mother but also resistance against the threat of hazardous microorganisms. Infection with pathogenic microorganisms during pregnancy may lead to premature delivery, miscarriage, growth restriction, neonatal morbidity, and other adverse outcomes. Moreover, the host also has an intact immune system to avoid these adverse outcomes. It is important to note the presence of normal bacteria in the maternal reproductive tract and the principal role of the maternal-placental-fetal interaction in antimicrobial immunity. Previous studies mainly focused on maternal infection during pregnancy. However, this review summarizes the new views on the study of the maternal microbiome and expounds the innate immune defense mechanism of the maternal vagina and decidua as well as how cytotrophoblasts and syncytiotrophoblasts recognize and kill bacteria in the placenta. Fetal immune systems, thought to be weak, also exhibit an immune defense function that is indispensable for maintaining the safety of the fetus. The skin, lungs, and intestines of the fetus during pregnancy constitute the main immune barriers. These findings will provide a new understanding of the effects of normal microbial flora and how the host resists harmful microbes during pregnancy. We believe that it may also contribute to the reference on the clinical prevention and treatment of gestational infection to avoid adverse pregnancy outcomes.

## Introduction

In recent years, research on the relationship between intestinal flora and diseases has become an interesting field, mainly focusing on the relationships between intestinal flora and liver disease, tumor, encephalopathy, autoimmune diseases, reproductive health and so on (1–3), and studies in this field imply that transplanting gut bacteria could be a new way to prevent and treat some diseases (4). For example, *Streptococcus lactate*, which plays a role in innate immunity to the reproductive system, can reduce uterine inflammation by regulating the balance between pro-inflammatory and anti-inflammatory cytokines (5). However, few studies have been conducted on normal microbiome, and the protective mechanism of the maternal, placental, and fetal immune systems against microbial infection during pregnancy is unclear.

Pregnancy is regarded as the process of growth and development of the fetus, which has the father's antigens, in the womb. In this process, the fetus not only needs to be resistant to maternal rejection but also must cope with challenges from the external environment. Several studies have already confirmed that microbial infections during pregnancy, such as bacteria, fungi, or viruses, are important risk factors for adverse pregnancy outcomes, including recurrent miscarriage, eclampsia, intrauterine growth retardation, premature rupture of membranes, and premature delivery (6, 7). In such a “dangerous” environment, a rapid and accurate immune response to pathogenic microorganisms is required for a successful pregnancy. Additionally, a successful pregnancy depends on the collective immune regulation of the mother, placenta, and fetus (8, 9). This paper will describe features of the normal microbial community during pregnancy and mainly focus on the antimicrobial immune mechanisms from the mother, placenta, and fetus during pregnancy. These conclusions are expected to provide new thoughts for the clinical treatment of infective disease during pregnancy.

## Normal Microbiome Of The Reproductive Tract During Pregnancy

The proportion of various microbiota in the female reproductive tract is up to ~9% of the total bacterial load in humans (10). As we know, the female genital tract is divided into upper genital tract, which is regarded to be bacteria-free, and lower upper genital tract, the former includes ovary, fallopian tube, and uterus, and the latter consists of the cervix and vagina. At present, the consistent results illustrate that *Lactobacillus* is the most in the uterine cavity, followed by *Pseudomonas* and *Acinetobacter* (11). Nevertheless, *Acinetobacter* is more abundant than *Comamonas* and *Pseudomonas* in the fallopian tubes (12). The classification of reproductive tract bacteria in pregnant women was further elucidated (Table 1). Recently, the development of transcriptomics, proteomics, and metabolomics has greatly improved research on the microbiome. Amy McMillanma, using multiplatform metabolomics analysis, showed that the normal vaginal flora in pregnant women is composed of *Lactobacillus crispatus, Lactobacillus iners, Gardnerella, Prevotella, Sneathia, Atopobium, Dialister*, and *Megasphaera* species (13). A longitudinal high-throughput pyrosequencing assay of the 16S RNA genes of the entire vaginal flora of normal pregnant women indicated that the flora was stable throughout pregnancy (18). However, during delivery, the amount of lactobacilli begins to decline, and the diversity of other vaginal flora increases; as a result, the vaginal microbial flora during delivery is more similar to that of non-pregnant females than that during pregnancy (19). Genetic sequencing was used to detect the vaginal microbiome in 1,958 pregnant women during the first and second trimesters of pregnancy (20). Consistent concepts also confirmed that preterm labor is due to a decrease in lactobacilli, rather than an increase in other microbiomes (21, 22). These results indicate that the amount of *Lactobacillus vaginalis* can be a clinical tool to forecast the risk of preterm labor (20).

**Table 1:** Normal microbiome of reproductive tract during pregnancy.

**Location**	**Technology**	**Main flora**	**Sample (*n*)**	**References**
Vagina	Multi-platform Metabolomics	*L. crispatus* *L. iners L. gasseri*	131	(13)
Cervical	DNA sequencing	*Lactobacilli* *Gardnerella*	10,049	(14)
Placenta	DNA sequencing	*E. coli* *Prevotella tannerae* *Bacteroides* spp.	57	(15)
Amniotic fluid	16S rRNA gene sequencing	*Streptococcus* spp *F. nucleatum*	48	(16)
Umbilical cord blood	16S RNA gene	Genus *Enterococcus Streptococcus* *Staphylococcus*	20	(17)

The stability of the vaginal microflora is also affected by different factors. *Lactobacillus* species are one example. *L. crispatus* plays a key role in maintaining the stability of the vaginal environment throughout pregnancy; however, if *Lactobacillus gasseri* and/or *L. iners* dominate during the first trimester, then they induce abnormal vaginal bacterial conditions after the third trimester (23). Nasioudis et al. evaluated relative abundance of bacteria in the vaginal microbiome in first-trimester pregnant women, and the results showed that *L. crispatus* was the numerically most abundant bacterium in 76.4% of women with a first conception, 50.0% with only a prior spontaneous or scheduled abortion, and 22.2% with a prior birth; *L. iners* was the most abundant bacterium in 3.8% of women with a first conception as compared to 19.2 and 20.8% in those with a prior abortion or birth, respectively; *Gardnerella* as the most abundant bacterial genus increased from 3.8% in women with a first conception to 15.4 and 14.3% in those with a prior abortion or birth, respectively (24). High estrogen during pregnancy is also another factor because a high estrogen level can induce *lactobacilli* to more efficiently utilize the vaginal epithelium to decompose glycogen and lactic acid, and a low vaginal pH is optimal for *lactobacilli* and eliminates the invasion of other harmful bacteria (24, 25). Therefore, Gjerdingen et al. claim that vaginal pH can be a predictive index of vaginal infection in pregnant women (26). There are few studies on the microbiome of the cervix during pregnancy. One result revealed that the cervical microbiome is analogous to the vaginal microbiome and that it mainly consists of *Lactobacillus* and *Gardnerella* (14). In the later stages of pregnancy, the cervical microbiota is likely to be similar to that of non-pregnant women (27). A large number of female reproductive tract bacteria were tested by 16S RNA and cell culture techniques. The results suggested that *Lactobacillus* is dominant in the uterine cavity, followed by *Pseudomonas* and *Acinetobacter*. Nevertheless, *Acinetobacter* is more abundant than *Comamonas* and *Pseudomonas* in the fallopian tubes (28).

The conventional concept that the placenta is sterile has been challenged (29, 30). In fact, Aagaard et al. tested 320 (normal pregnancy:complication of pregnancy) anatomical placenta with 16S ribosomal DNA–based and discovered that there is a low abundance but metabolically rich microbiome in placenta: *Firmicutes, Tenericutes, Proteobacteria, Bacteroidetes*, and *Fusobacteria phyla*, which are positive correlation with early abortion (31). Besides, these bacteria are similar to those found in the mother's mouth and may be transmitted through blood (12). Recently, another experiment with the same technology explained deeply that the matrix side of the placenta is mostly *Ralstonia*, an aerobic o-film-forming bacillus, while *L. iners* and *L. crispatus* are located on the fetal side of the placenta (15). However, the idea has been refuted by de Goffau et al. who demonstrated that human placenta has no microbiome but can contain potential pathogens, which was published in Nature this year. Their team collected placental biopsies from a total of 537 women, including 318 cases of adverse pregnancy outcome and 219 controls, and checked with both metagenomics and 16S amplicon sequencing, then elaborated three significant results, as follows: first, the biomass of bacteria is very low, nearly too clean; second, the reason why someone thinks that placenta has microbiome is that the samples were contaminated during labor or operation; and last, there is no obvious connection between microbes of the placenta and preeclampsia or recurrent abortion (32).

In addition, there are also some flora in the umbilical cord blood, amniotic fluid, and amniotic membrane. The genera *Enterococcus, Streptococcus, Staphylococcus*, and *Propionibacterium* have been detected in umbilical cord blood (17), and *Streptococcus* spp. and *Fusobacterium nucleatum* are present in amniotic fluid, which may be derived from the mother's mouth (16). Additionally, the maternal milk contains staphylococci, streptococci, bifidobacteria, and lactic acid-producing bacteria; these acid-producing *Lactobacillus* bacteria may be transported from the mother's intestine through the blood to the breast milk and then into the fetal intestine (33).

Currently, researchers are increasingly interested in the transition of the vaginal flora of the mother to the intestinal flora of the fetus. Dominguez's team discovered that the flora of infants delivered vaginally were similar to that of the mother's vagina; however, infants delivered by cesarean section have flora similar to that of the mother's skin (34). In recent years, clinical studies have confirmed that the intestinal flora of infants born by cesarean section became analogous to that of infants born vaginally after 30 days of the continuous application of vaginal fluid to the anus of the infant. This finding suggests that the maternal vaginal flora results in a certain regulatory effect on the infant's intestinal flora (35). At the same time, the maternal vaginal microbiome also has a certain influence on the growth and development of the fetus. Martinez et al. showed that compared with mice born by vaginal delivery, mice born by cesarean section lacked dynamic changes in intestinal microflora, which led to weight gain after weaning (36). This result demonstrates that maternal vaginal bacteria are relative to the normal metabolism of the fetus and affect its growth and development (36). This transfer of maternal flora to the infant may be caused by the metabolites of microbial molecules, and it may influence the development of the innate immune system of the newborn (37).

## Characteristics AND Outcome Of Infection During Pregnancy

If a mother is infected by microorganisms during pregnancy, inflammation will cause clinical signs and symptoms, such as fever, diarrhea, and abdominal pain. On the other hand, a more important fact is the effects of inflammation on pregnancy outcomes, such as increased risk of miscarriage, premature birth, and stillbirth (6). Even if the virus does not reach the fetus, the level of the maternal inflammatory response and the levels of inflammatory cytokines, such as interleukin (IL)-1, IL-6, IL-8, and tumor necrosis factor (TNF)-α are very high, which can affect the development of the fetal brain and circulatory system, and may increase the risk of schizophrenia, autism, and mental disorders (38). Moreover, it should be noted that approximately half of pregnant women who are infected without showing infectious symptoms give birth prematurely, which may be related to past placental infections or acute and chronic chorioamnionitis (39).

Epidemiological and microbiological studies show that 25–40% of premature births are caused by intrauterine infection (40). It is worth noting that the risk of infection by some pathogenic microorganisms is not limited to adverse pregnancy outcomes but the abnormal development of various organs in infants. For example, in the clinic, the most common TROCH infection, including *Toxoplasma gondii*, rubella virus (RV), cytomegalovirus (CMV), herpes simplex virus (HSV), and other viruses, can result in premature birth, stillbirth, and even neurological disorders after birth (41, 42). On the other hand, the risk of microbial infection in the fetus and the severity of the disease depend on the stage of pregnancy. For instance, RV infection during the first stage may cause abortion and congenital malformation. However, as pregnancy progresses, in the middle and late stages of pregnancy, the rate of congenital malformation caused by the RV is very low. CMV is most likely to infect a fetus during the third trimester of pregnancy, whereas the greatest damage occurs in the first trimester. The phenomenon referred to above may be due to differences in the growth state of the fetus and its ability in resisting the external environment during diverse pregnancy periods (42).

Infections during pregnancy can also cause a variety of health problems in the fetus after birth. Researchers from the University of Massachusetts Medical School and Massachusetts Institute of Technology conducted a study of gut microbes related to fetal autism. They found that intestinal microbial infections in pregnant women can activate immune cells to secrete a large amount of IL-17?, which passes through the placental barrier and enters into the fetus, forming “plaques” in the S1DZ region of the fetal brain. As a result, this abnormality can influence the development of the fetal central nervous system, causing diseases, such as autism (43).

## Resistance To Pathogenic Microorganisms During Pregnancy

### Defense Mechanism of Maternal Vagina and Decidua

#### The Innate Immunity of the Lower Genital Tract

**Figure 1:**
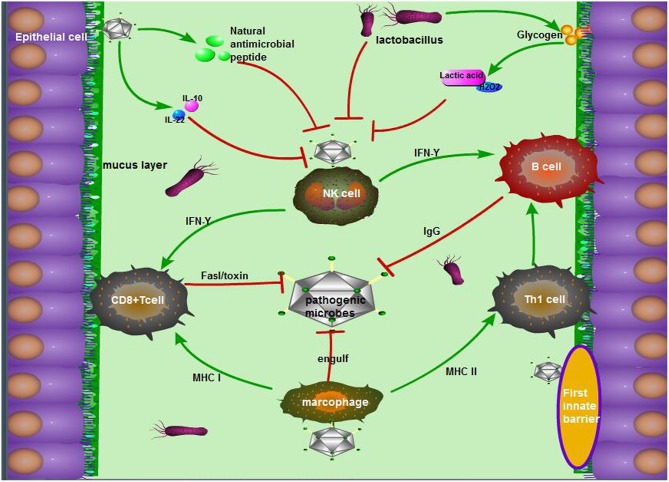
The antimicrobial mechanism of the vagina during pregnancy. The mechanism mainly consists of the following three parts: (1) The first barriers: tight junctions of epithelial cells, movement of microvilli, and the package of mucus, as well as natural antimicrobial peptides from epithelial cells; (2) Exclusive effect of *Lactobacillus* on other pernicious biota: lactic acid, acidolin, lactacin, H_2_O_2_; (3) The pathway including innate immune cells in the vagina, innate immune response, and acquired immune response. To be exact, antigen-derived cells, which make a sense by innate immunity, present pathogen antigens to acquired immune cells to kill, and crack pathogens.

The innate immune system of the vagina is mainly composed of a tissue barrier, immune cells, and innate immune molecules (Figure 1). Doerflinger et al. established a three-dimensional (3-D) vaginal model, expounding the composition of the vaginal tissue barrier, which includes flat epithelial cells, tight connections, microvilli, microbridges, secreted mucus, and so on (44). The main function of epithelial cells is to recognize microbial antigens through toll receptors on the surface, thereby activating nuclear factor (NF)-κB and subsequently stimulating the production of pro-inflammatory factors (45). Close connections and microvilli perform the function through isolating harmful microorganisms (46). When the vagina is infected, viral dsDNA can increase the secretion of membrane-associated vaginal mucus in various ways, and this mucus can not only lubricate the vagina but also adhere to pathogens to fight against the microorganisms (47). The antigen-presenting cells, macrophages, and natural killer (NK) cells in the lower genital tract can also use their pattern recognition receptors [such as Toll-like receptors (TLRs), c-type lectin receptors, and NOD receptors] to combine with different bacterial products and transmit inflammatory signals in the cell, activating many kinds of T cells, and increasing the IgA antibody levels of plasma cells from B cells. Ultimately, the vaginal mucosal immunity remains stable in a safe range (48). In the meantime, antimicrobial peptides, cytokines, and chemokines participate in the innate immunity of the vagina through the pathogenic molecular model (49). The antimicrobial peptides secreted by epithelial cells exert a variety of functions in the lower reproductive tract mucosal immunity, including preventing the invasion of microorganisms into host cells, regulating immunity, inhibiting inflammation, and maintaining the stability of the internal environment (50). Cationic antimicrobial peptides can neutralize and lyse bacteria by binding with bacterial anions (51). These peptides can kill fungi by binding to specific receptors (52). For viruses, antimicrobial peptides attack the glycoproteins on the surface of the virus to prevent the virus from attaching to host cells (53).

During pregnancy, many factors may influence the state of the vagina's immunity to pathogens. For example, changes in the morphology of the vaginal mucosa, the regulation of the mucosal environment, and the hormonal level in the female reproductive tract are essential to resist external pathogens (54). Faure et al. found that the vagina could produce an anti-inflammatory response (a 5-fold increase of IL-10) and a strong mucosal homeostasis response (a 40-fold increase of IL-22) to fight BV infection by detecting the vaginal tissue samples of pregnant women infected with BV (55). However, IL-22 participates in mucosal homeostasis and avoids invading commensal bacteria by inducing the synthesis of mucosal antimicrobial peptide and mucus, promoting wound repair and reinforcing epithelial tight junctions (56). In addition, the normal vaginal microbiome also plays an indispensable role in maintaining vaginal homeostasis. The acidolin, lactacin, and H_2_O_2_ produced by lactobacilli were capable of increasing the activity of host antimicrobial peptides (muramidase and lactoferrin) and consequently enhanced the antibacterial activity of epithelial cells (57). As Doerflinger et al. noted, during pregnancy, lactobacilli could cause vaginal epithelial cell activation and minimally disrupt immune barrier properties, which indirectly suggests its role as a beneficial species vaginal microbiome (44).

#### The Immunity of the Decidua

After implantation, the endometrium is called the uterine decidua and is where a number of immune cells are located, such as NK cells (70–80%), macrophages (20–25%), T cells (<2%), dendritic cells (DCs) (<1.7%), and granulocytes and B lymphoid cells at the lowest levels (58). The most abundant cells in the decidua, the NK cells, mainly produce interferon (IFN)-γ to participate in the following immune response mechanisms: (1) macrophages and neutrophil cells were stimulated by IFN-γ to induce the production of antimicrobial peptides; (2) IFN-γ can increase the secretion of IL-1 from T cells to play a role in anti-inflammation; and (3) IFN-γ can stimulate B lymphocytes to differentiate into plasma cells and secrete antibodies (58). When HCMV infects the mother, it will induce a rapid and strong decidual tissue innate immune response, which is caused by IFN-γ from NK cells, IP-10 from DC cells, and the downregulation of decidual cytokines/chemokines in a unique way (59). However, the elimination of virus does not always depend on the activation of decidua immune cells. Last year, a group of analyses with whole-genome transcriptomes concluded that ZIKV did not induce the activation of decidua immune cells but reduced the replication of the virus by causing apoptosis and the death mechanism of infected cells (60). Further study elucidated that the immune cell phenotypes of macrophages will also affect their antimicrobial status during pregnancy (the phenotypes of macrophages are M1 and M2). M1 macrophages play an anti-inflammatory and antimicrobial role through common phagocytosis and antigen-presenting modes, whereas M2 macrophages regulate the immune system during pregnancy (61).

The immune function of the decidua is caused not only by its immune cells but also by common natural antimicrobial peptides. When the pattern recognition receptor on the endometrium is activated, the expression of antibacterial peptide transcripts will be upregulated to help the uterine decidua produce an immune response to infection (62). Two major antimicrobial peptides were found in the decidua: defensins and whey acidic protein (WAP) motif proteins (63). Defensins have six cysteine residues that form three disulfide bonds and are divided into two main groups on the basis of the position of these bonds, β-defensins and α-defensins (64). These defensins are considered a medium to recruit monocytes, T lymphocytes, and DCs (65). The WAP motif protein family includes secretory leukocyte protease inhibitor (SLPI), Trappin-2/elafin, eppin, and HE4. Apparently, these molecules have multiple functions, including direct antimicrobial activity, bacterial opsonization, adaptive immune response induction, and tissue repair promotion (66). Among these WAP proteins, SLPI and elafin are the most widely studied (67). These two proteins can be continuously expressed by uterine decidua cells, and their expression level is affected by the proinflammatory environment, neutrophil elastase, and sex hormones (68). Jin et al. showed that SLPI could suppress the lipopolysaccharide (LPS)-induced activation of NF-κB and reduce the synthesis of TNF-α/nitric oxide, thus inhibiting the inflammatory response (69). When elafin resists some viral infections, such as HIV, there is a direct interaction between them, but the detailed mechanisms are still poorly understood (70). Other antimicrobial peptides involved in the innate immunity of the uterine decidual membrane have been detected, for example, HBD-3, CCL20/MIP-3a, and lysozyme (63, 71, 72).

### The Function of the Placental Barrier

#### TLR Pattern on the Placenta

The placenta performs a diverse range of essential functions during pregnancy, including the exchange of gases, nutrients, and metabolites between the mother and fetus, the secretion of hormones, and acting as a physical immune barrier between them (59). TLR is a protein mainly expressed in mononuclear macrophages, other lymphocytes, and epithelial cells (73). Zarember and Godowski have discovered the existence of 10 types of TLRs in human placenta (74): TLR1, TLR2, TLR4, TLR5, TLR6, and TLR10 are expressed on the cell surface to identify the microbial membrane components of gram-negative bacteria and gram-positive bacteria, whereas TLR3, TLR7, TLR8, and TLR9 exist in the cell to identify single nucleic acid molecule, diphosphonic acid, and molecular RNA and to remove apoptotic fragments from cells. In addition, these receptors have a regular expression in space and time in the placenta. TLR2 and TLR4 are mainly present in the placental villi and trophoblasts (75, 76), and the expressions of TLR2, TLR3, and TLR4 are significantly decreased in early pregnancy (77). These facts indicate that the activation of TLRs on the placenta may have multiple effects, including immune cell recruitment, cytokine secretion, and protective responses to invading pathogens (78).

#### The Antimicrobial Role of Trophoblast Cells and Syncytiotrophoblasts

Multipotent trophoblast progenitor cells (TBPCs) were reported to reside in the chorion of the human placenta and differentiate into mature trophoblast subtypes, namely, transport syncytiotrophoblasts, and invasive trophoblast cells (also known as cytotrophoblasts), both of which continuously proliferate to form the functional unit of the placenta (79). The immunomodulatory effects of trophoblast cells on the maternal-fetal interface are irreplaceable, including the recognition of bacteria and viruses and the recruitment of leukocytes to respond to pathogens (80). Previous experiments have verified that the trophoblast recognizes pathogens through TLRs, such as TLR-3, TLR-7, TLR-8, and TLR-9, and this interaction leads to the production of cytokines and chemokines that regulate leukocyte migration to eradicate the pathogens that cause infection (81). Epithelial cadherin (e-cadherin) is a receptor expressed in the cytotrophoblast layer that can specifically recognize *Listeria* endotoxin A to restrain the spread of this bacterium (82). As for the virus (CMV, *Toxoplasma gondii*, and *Porphyromonas gingivalis*), immune trophoblast cells can bind to poly (a special marker of most viruses) by TLR-3, promoting the secretion of SLPI and IFN-γ against viruses, which ultimately prevents the virus from spreading from the placenta to the fetus (83). However, decidual trophoblasts can also secrete CXCL12 (SDF1), CXCL8 (IL-8), transforming growth factor (TGF)-β1, and CCL2 (MCP1) to recruit macrophages, NK cells, and regulatory T (Treg) cells, representing a connection between innate immunity and acquired immunity (8).

**Figure 2:**
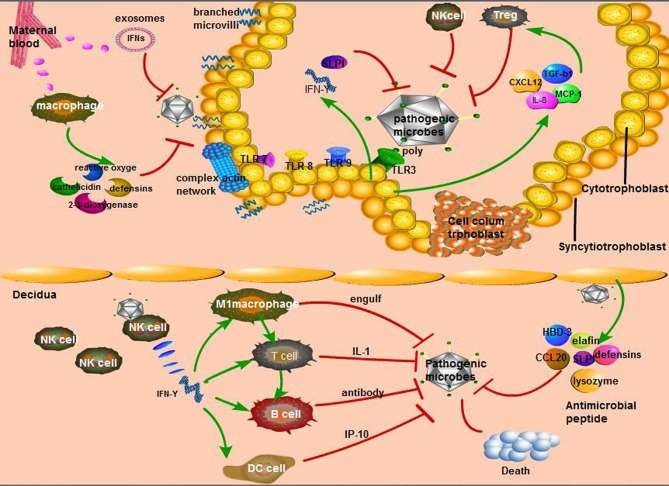
Unique way of maternal-fetal interface resistance to microorganisms. (1) The main immune response in the maternal decidua: interferon (IFN)-γ secreted by natural killer (NK) cells, the most affluent cells in maternal decidua, recruits other immune cells to perform active function. Second, various antimicrobial peptides secreted by decidua cells can resist pathogenic microorganisms; (2) The cytotrophoblast relies on its diverse Toll-like receptors (TLRs) to recognize bacteria, fungi, and so on, then causes the secretion of diversity anti-inflammatory factors that activate specific immune cells; (3) The defensive role of syncytiotrophoblast is mainly from the hardness of its cells consisting of branching microvilli and complex actin network as well as maternal macrophages.

Fetal syncytiotrophoblasts are located at the periphery of the cytotrophoblast layer, forming a unique fused polynuclear surface that is infiltrated into the maternal blood. Since syncytiotrophoblasts are attacked by pathogens circulating in the maternal blood, these cells are likely to have distinct mechanisms to resist microbial invasion, including *T. gondii, Listeria monocytogenes* (84), and viruses such as ZIKV, HSV, CMV, which may contribute to the absence of receptors (85). The surface of the syncytium has unique physical properties, such as dense branched microvilli on the apical surface and a complex actin network on the cortex (84). In addition, the young index of syncytiotrophoblasts is 1.5–4.5 times that of red blood cells in hemolytic anemia patients, indicating a high level of hardness that prevents microbial invasion through the trophoblast layer, which is known as the physical barrier (85). Moreover, macrophages in the maternal blood are attracted to microorganisms, which then produce high levels of 2,3-dioxygenase, β-defensins, reactive oxygen species, cathelicidin, and nitrogen responsible for the extracellular trophoblast resistance to pathogens (63). *Listeria* infection can induce the defense of syncytiotrophoblasts in the first stage of pregnancy, which is mediated by the transport of placental exosomes carrying miRNA and IFNs (84). The pathways of maternal-fetal interface resistance to microorganisms were shown in Figure 2.

The placenta, of course, not only has an innate immune system but also its own acquired immunity. We demonstrated that maternal CD4+ T cells play an important role in protecting the maternal immune responses against fetal death. The production of CD4+ T-cell cytokines and the interaction between CD4+ T cells and antigen-presenting cells stimulate the proliferation of the cytotoxic CD8+ T-cell population, and then the cytotoxic cells can clear the virus-infected cells through the fas-fasl pathway (86). Moreover, CD4+ and CD8+ T cells are also involved in host response to *Toxoplasma* infection (87).

Girardi et al. found that the complement system is activated at the placental interface during early and late pregnancy (88). Moreover, Goldberg et al. discovered eight complement cytokines in villi, including the factors B, C3, C1r, C1s, and C1 and the inhibiting factors H, C4, and C2 (89). Further research showed that C3 and C4 are mainly expressed by trophoblastic cells and that IFN-γ can increase their expression. The secretion of these complements improves the defensive function of the placenta (80).

### The Shield of Amniotic Fluid

Amniotic fluid is known to be the protective fluid that surrounds the fetus throughout pregnancy, providing an internal environment for fetal growth and development (90). Diverse immune cells form a solid barrier (91). Cytological studies have shown that amniotic fluid contains many innate immune cells, including macrophages, neutrophils, and innate lymphocytes (ILCs) (92, 93). Additionally, Gomez-Lopez et al. collected amniotic fluid from 57 females with normal pregnancies at multiple centers in the United States and noted that T cells and intrinsic lymphocytes (ILCs) were the most abundant cells, more abundant than B cells and NK cells, at 15–30 weeks of gestation. B cells and macrophages were scarce before 20 weeks of gestation and would increase after 20 weeks and remained stable until delivery. It is surprising that most of these immune cells come from the fetus (94).

### Fetal Self-Protection Against Pathogenic Microorganisms

It is acknowledged that the fetus is highly sensitive to infectious antigens, especially in the first 3 months of pregnancy due to various exposures and fetal memory loss to immunity. Thus, the fetus can only depend on innate immunity against microbial infection (95). Dasari et al. reported that TLRs are expressed in fetal monocytes and granulocytes in the same way as adults. In addition, the phagocytic function of NK cells, macrophages, and DCs is similar to that of adults, but their antigen presentation ability is very weak (96). Based on a large number of studies on ZIKV in recent years, Chen et al. found that fetus-derived IFN-I signaling contributes to the anti-ZIKV response and that this signaling pathway involves the potential antiviral activators Janus kinase (Jak1 and Tyk2) and signal sensor (STAT1 and STAT2), resulting in the upregulation of hundreds of IFN-stimulated genes (ISGs) (97). Current research shows that fetal acquired immunity is weak and mainly comes from the mother. After 22 weeks of gestation, this transmission soars, and IgG levels exceed maternal levels at birth (98).

The lung, gastrointestinal tract, and skin are frequently exposed to amniotic fluid during pregnancy and are targets of infection. At the time of skin infection, epidermal keratinocytes start to synthesize peptides, such as cathepsin, which has the ability to inhibit bacterial growth or destroy bacteria, resulting in a rapid increase in volume (99). It has been proven that chorioamnionitis induces the upregulation of TLR2 and TLR4 and a rise of cytokines, chemokines, and antimicrobial factors in epidermal keratinocytes (100, 101). The sentinel immune cell of the lung is the alveolar macrophage, and chorioamnionitis can trigger the production of alveolar macrophages through fetal immunity; moreover, IL-6, which stimulates placental secretion, not only activates type II alveolar cells but also increases SP-A synthesis to promote lung maturation; thus, further enhancing fetal lung immunity (102). M cells are the first protective cell layer of gastrointestinal defense. Additionally, the lamina propria also contains a variety of immune cells, such as DCs and macrophages in the lamina propria of the intestinal epithelium (103). When fetal intestinal epithelial cells are exposed to bacterial LPS antigens, the condition can also stimulate strong expression of IL-8, thus recruiting DCs and macrophages to play an innate immune barrier role (104).

### Efficient Immunity to Virus Infection During Pregnancy

As mentioned above, severe viral invasion during pregnancy can lead to adverse outcomes, and antiviral immunity depends on the mother instead of the fetus whose acquired immunity is immature. The mucosal layer of the female lower genital tract can effectively prevent the entry of the virus (105), and the low PH can also resist it to some extent (106). Akiko Iwasaki's lab considers TLR as an important recognition to viral, for instance, TLR3 can quickly recognize the HSV (107). Decidual NK (dNK) whose major subset is CD56^bright^ CD16^neg^ might be an important component of the local innate immune response to uterine virus infection through specific ligand triggering of NKp46 during early pregnancy (108). Mandelboim et al. discovered further that some virus-infected cells might be recognized by the NKp46 receptor through the binding of viral hemaglutinin, inducing a rise of intracellular calcium mobilization, perforin polarization, granule exocytosis, and efficient target cell lysis (109). Meantime, the study has proven that if the dNK cells are exposed to HCMV, it may happen with phenotypic changes and the acquisition of a cytotoxic function involving the NKG2D activating receptors (similar to NKp46) to prevent viral spread and placental pathology (110). dNK cells can not only prevent congenital HCMV infection by reducing the secretion of relative to trophoblast invasion such as CCL2, CCL4, CCL5, CXCL10, granulocyte–macrophage colony-stimulating factor, and CXCL8 but also activate other immune cells, namely, T cells (111). Additionally, it is reported with murine CMV model that E3 LigaseTRIM29 is a critical checkpoint regulator of NK cell functions and plays an important role in host defense against CMV infection, therefore, TRIM29 may be a good target for controlling CMV infection and spreading during pregnancy (112). For innate antiviral from trophoblast cells and syncytiotrophoblasts, as mentioned above, the former can depend on the identification of TLR-3 to viral followed by the secretion of SLPI and IFN-γ (83), the latter relies on unique physical properties, such as dense branched microvilli on the apical surface and a complex actin network on the cortex (84).

When it comes to acquired immunity to virus, which mainly involves memory B cells, memory CD8 ± T cells, and memory T cells, then their team continues to study that circulating memory B cells are recruited to the vaginal mucosa in a CXCR3-dependent manner and secrete virus-specific IgG2b, IgG2c, and IgA that can be transported by FcRn into the vaginal lumen as a result of a rapid and strong immune defense response (113, 114). On the other hand, memory CD8± T cells in the vaginal mucosa are considered to be the most important immune cells against virus (115), histocompatibility complex class I (MHC I) transited by CD301b± DCs, and NK cells can stimulate CD8± T to secrete IFN-γ, which can resist viruses (116). This response can be enhanced by DCs and B cells (117). Additionally, CD4 tissue-resident memory T cells can also be stimulated and secrete IFN-γ, which causes expression of chemokines, including CXCL9 and CXCL10 (113). There is a specific example about IFNs resisting virus, that is, ZIKV, which can be transmitted sexually between humans. Vaginal infection of pregnant dams during early pregnancy led to fetal growth restriction and infection of the fetal brain in WT mice (118). Type I IFNs are essential for host resistance against ZIKV, and IFN-α/β receptor (IFNAR)-deficient mice are highly susceptible to ZIKV infection (119). Yockey et al. revealed that after ZIKV infection, IFNAR signaling in the conceptus inhibits development of the placental labyrinth, resulting in abnormal architecture of the maternal-fetal barrier (120). Their results implicated type I IFNs as a possible mediator of pregnancy complications, including spontaneous abortions and growth restriction, in the context of congenital viral infections (120).

## Concluding Remarks and Future Perspectives

Generally speaking, there is no intrauterine sterility during fetal growth and development. An increasing number of commensal flora in the reproductive tract of pregnant women have been discovered, which can limit the intrusion of deleterious bacteria to some extent. However, the mother, placenta, and fetus also possess unique innate immune systems. The immunity of the mother mainly depends on the lower genital tract and decidua, which consist of the balance between common bacteria groups and the mucosal barrier. In addition, trophoblasts and syncytiotrophoblasts have meaningful functions in the immunity of the placenta, such as the role of immune cells, the villi barrier, complement system, and so on. The fetus can utilize its immature specialized organs and network of cytokines to protect itself.

It is acknowledged that to prevent maternal infection and adverse neonatal outcomes during pregnancy, clinical medication is commonly used, although the optimal formulation and dosage of most drugs have not been described specifically for pregnant women (121). For Group A streptococci infection or other Galanz-positive bacteria, clindamycin has more advantages compared to penicillin because of a longer post-antibiotic effect and less side effects (122). However, a meta-analysis including 15 trials (involving 1,754 women) suggests that amoxicillin and clindamycin show less side effects and more effective results than azithromycin and erythromycin in the way of genital *Chlamydia trachomatis* infection during pregnancy (123). For the treatment for virus, the administration of high-avidity immunoglobulin (HIG) to prevent maternal-fetal CMV transmission is a must in the first trimester pregnancy (124). After transmission, administration of HIG can also be considered to either prevent a symptomatic infection at birth or lessen the symptoms (125). The result from Ville et al. indicates that high-dosage valacyclovir given is effective for improving the outcome of moderately symptomatic infected fetuses for virus infection (126). For these reproductive tract virus infection, clinical immunotherapy-vaccine has become a major prevention, for example, the HSV vaccine, which can reduce recurrence rate of HSV by ~50% (127). Wald A even utilized Helicase-primase inhibitor pritelivir compared with valacyclovir in a randomized controlled clinical study to intervene HSV vaginal infection in the result of decreasing clinical recurrence and shedding rate by 99% (128, 129). According to the latest research, taking advantage of the CD8± T resistance to HSV virus effectively in the vagina, David demonstrated in an animal model that imiquimod (inducing CD8± T cells) combined vaccine can significantly reduce the recurrence of HSV, which is expected to be applied in clinical practice (116). What is more, all kinds of vaginal antimicrobial peptides and symbiotic bacteria mentioned above can boost the innate immunity against vaginal viruses (such as HIV, HSV, HPV, etc.) to a large extent (130, 131).

Healthy pregnancy requires tightly coordinated immune responses. When dysregulated or inappropriately expressed, these have the potential to act as teratogens and disrupt fetal and placental developmental pathways, leading to birth defects and pregnancy complications (132). With the emergence of new pathogens and our further understanding of pathogens during gestation, evaluating and treating gestational infections will require a more accurate and effective evaluation system. This review will support a deeper and more profound comprehension of microbiological immunity during pregnancy and provide a theoretical resource for clinical implementation. In future studies, we should consider the pathological mechanism of gestational infection and focus on how to design more accurate biomarkers to detect subclinical infections to avoid the adverse and long-term effects of pathogenic microorganisms on the growth of children. In other words, solidifying the ability of the maternal immune to ensure successful gestation is important.

## Author Contributions

CM was responsible for writing the manuscript. WY finished in the translation work. XW completed the production of charts and figure. KW made the proofreading. DH designed and revised the manuscript.

### Conflict of Interest

The authors declare that the research was conducted in the absence of any commercial or financial relationships that could be construed as a potential conflict of interest.
